# Detection of Selection Signatures in Chinese Landrace and Yorkshire Pigs Based on Genotyping-by-Sequencing Data

**DOI:** 10.3389/fgene.2018.00119

**Published:** 2018-04-09

**Authors:** Kai Wang, Pingxian Wu, Qiang Yang, Dejuan Chen, Jie Zhou, Anan Jiang, Jideng Ma, Qianzi Tang, Weihang Xiao, Yanzhi Jiang, Li Zhu, Xuewei Li, Guoqing Tang

**Affiliations:** Farm Animal Genetic Resources Exploration and Innovation Key Laboratory of Sichuan Province, Sichuan Agricultural University, Chengdu, China

**Keywords:** pig, selection signature, artificial selection, domestication, genome

## Abstract

The domestic pigs have been undergone intense selection pressures for these development of interested traits following domestication and modern breeding. This has altered many traits in most of pig breeds, such as growth rate, body weight, fertility, and immunity. Thus, the objectives of this study were to (1) detect these selection signatures and identify the candidate genes that show evidences of recent artificial selection at the level of whole genome, (2) be beneficial to understand the relationship between genomic structure and phenotypic diversity, and (3) highlight the key roles of these candidate genes in growth and development in the two breeds. The data consisted of total raw number of 345570 single nucleotide polymorphisms (SNPs) in 1200 individuals from the Chinese Landrace pigs (L, *n* = 600) and Yorkshire pigs (Y, *n* = 600). Based on these SNPs data, two complementary methods, population differentiation (Fst) and composite likelihood ratio test (CLR), were carried out to detect the selection signatures in this study. A total of 540 potential selection regions (50 kb) which contained 111 candidate genes were detected for Landrace-Yorkshire pair (L-Y) by Fst. In addition, 73 and 125 candidate genes were found for Landrace pigs and Yorkshire pigs by CLR test based on 321 and 628 potential selection regions, respectively. Some candidate genes are associated with important traits and signaling pathways including the *ACACA*, *MECR*, *COL11A1*, *GHR*, *IGF1R*, *IGF2R*, *IFNG*, and *MTOR* gene. The *ACACA* and *MECR* gene are related to fatty acid biosynthesis. The *COL11A1* gene is essential for the development of the normal differentiation. The *GHR*, *IGF1R*, and *IGF2R* gene are significant candidate genes which play major roles in the growth and development in animals. The *IFNG* gene is associated with some aspects of immune response. The *MTOR* gene regulates many signaling pathways and signaling transduction pathway.

## Introduction

As a major protein source of humans, the swine is one of the most important livestock (Amaral et al., 2011; Wilkinson et al., 2013). The domestic pig originated from the wild boar (*Sus scrofa*) that were mainly distributed in Europe and Asia (Larson et al., 2005, 2007). Artificial selection altered the phenotypic traits of these pigs. Many pig breeds were objectively selected for desirable performance traits such as rapid growth, increased lean meat and enhanced prolificacy. A number of trait differentiations, therefore, occurred in a short time, resulting in the development of distinct pig breeds phenotypes. It is possible to investigate the effects of selective pressure on various livestock at the genome-wide level due to the development of high-throughput genotyping technologies. Studies have been concentrated on the specific traits relevant to the growth and development processes with the object of identifying and characterizing selection signatures, and then identifying the potential causal mutations in order to understand the genetic basis structure for phenotypic variation in the swine (Andersson, 2001; Andersson and Georges, 2004). Mutations conferring new favorable genotypes will be subject to a selective sweep, which is an increase rapidly in allele frequency under artificial selection. Moreover, the artificial selection led to the traits changes mainly related to growth and development. For example, a single nucleotide mutation in the *IGF2* gene lead to a major effect on muscle growth in some commercial pig breeds (Laere et al., 2003). The *MC4R* gene is related to growth and fatness both in pigs and humans (Kim et al., 2000; Ovilo et al., 2006).

When a beneficial mutation emerges and subsequently spreads in a population, this process, that is selective sweep (Smith and Haigh, 1974), will generate higher population differentiations, higher frequencies of segregating sites and linkage disequilibrium (LD) patterns (Grossman et al., 2010). In this study, the Fst and CLR test were carried out to assess population difference and detect artificial selection. Population differentiation was accessed by Fst based on the DNA polymorphism in populations (Wright, 1931; Hudson et al., 1992; Nielsen, 2005; Weir et al., 2005). While CLR test has a high power to detect selective sweeps using site frequency spectrum (SFS) patterns of SNP (Williamson et al., 2007).

Although many studies have been detected a lot of selection signatures in the pig, those findings are not entirely consistent. Andersson and Plastow (2011) and Rubin et al. (2012) detected selection signatures in European domestic pig and wild boar using whole-genome resequencing. Selective sweep analyses revealed strong selection signatures at three loci which were related to morphological changes in the domestic pig (Rubin et al., 2012). Fu et al. (2016) Identified 417 protein-coding genes which were mainly associated with developmental and metabolic processes by combining Enshi black pigs and Chinese wild boars. The reasons are not only the differences of the statistical methods, but also the varieties of SNP panels density and sample size. In addition, domestic pigs under different evolution conditions (different breeding objectives) show different selection signatures even in the same breed. Therefore, it is important to explore selection signatures in more pig breeds and these findings will provide a foundation to investigate the artificial selection process and domestication history of the two commercial pig breeds.

## Materials and Methods

### Genotype by Sequencing Data

In the study, we adopted a surfactant and the protease pyrolysis method to extract genomic DNA from ear tissue. A total of 600 Landrace and 600 Yorkshire pigs were genotyped using GBS technology (Elshire et al., 2011). The data were sequenced by using Illumina HiSeq PE150. In the raw reads, N contents with < 10% of sequence length or with high quality bases (>5) and a number < 50% of the sequence length were retained. The clean data were aligned against the Sscrofa 10.2 using Burrows Wheeler Aligner (BWA) with the parameters ‘mem –t 4 –k 32 –M’ (Li and Durbin, 2009). Then we found a total of 10,445,924 SNPs by using the Genome Analysis Toolkit (GATK) with default parameters (Depristo et al., 2011). And a total of 345,570 SNP markers met the quality requirements using VCFtools with the parameters ‘–min-meanDP 3 –maf 0.01 –max-missing 0.2′ (Danecek et al., 2011). The data that support the findings is publically available at figshare^1^ under doi: 10.6084/m9.figshare.5960914.

### Populations and the Data Quality Control

A total of 1200 individuals from two commercial pig breeds, 600 Chinese Landrace pigs and 600 Chinese Yorkshire pigs, were genotyped by sequencing. Genotyping contains raw data with a total of 345570 SNPs. And after quality control we obtained 92114 SNPs. The quality control was determined using the PLINK program (Purcell et al., 2007). A quality control was adopted to access the high data quality by (1) removing SNPs loci with call rate less than 0.95 and unknown position, (2) removing SNPs loci with minor allele frequency (MAF) less than 0.05, (3) discarding the individuals with call rate less than 0.95, (4) removing SNPs loci with Hardy Weinberg balance test less than 10-6 and (5) removing SNPs loci in sex chromosomes. Following the quality control, the missing genotypes was imputed using the BEAGLE (Browning and Browning, 2007). After imputation, the average estimated squared correlation (R2) between the allele dosage with highest posterior probability and the true allele dosage fore the marker is 0.9982. And then the principal component analysis (PCA) for population structure based on SNPs information was performed using the EIGENSOFT (Patterson et al., 2006).

### Methods for Detection of Selection Signatures

Two methods, Fst and CLR test, were implemented to detect the selection signatures. The two approaches are all directly handling the SNP genotype. Hudson’s Fst (Hudson et al., 1992), a classical measure of population differentiation, was used to detect selection signatures between Landrace pigs and Yorkshire pigs at each SNP in this study. For each SNP in a pairwise comparison, the expected Fst was calculated with the PopGenome v2.2.4 in R (Pfeifer et al., 2015).

A recent selective sweep changes patterns of allele frequency at linked sites, excluding variation at closely linked loci and generating a relative excess of allele at very low and very high frequencies at more aloof loci (Williamson et al., 2007). A statistical method, CLR test (Nielsen et al., 2005), was implemented to search for the particular pattern of allele frequencies along a chromosome following a selective sweep. The CLR value was computed using the SweeD program (Pavlidis et al., 2013) at each SNP for the two breeds respectively.

### Identification of Candidate Genes Under Selection and Gene Annotation

An outlier-approach to obtain the candidate genes under selection was taken (Yang et al., 2014). For all loci, a SNP which corresponded to the upper 1% of the empirical genome-wide distribution of Fst was considered to be a high-Fst outlier. Accordingly, candidate selected regions were deemed as the 99th percentile of the empirical genome-wide distribution of CLR. And then, the distance cutoff was limited to be 500 bp in order to obtain a high-quality gene for gene annotations. Thus, a SNP was judged to belong with a gene if it is located within the region defined by 500 bp upstream of gene start site and 500 bp downstream of the gene end site. Genes found within the intervals spanning the candidate regions were searched from the Ensembl genome browser^2^ using the Sscrofa 11.1 reference genome and these were considered as candidate genes. Function annotation and enrichment of the candidate genes were displayed using the DAVID browser^3^ and the KEGG^4^. Phenotype that are known to be affected by the identified candidate genes were inferred from the literature.

## Results

### Information of Filtered Data and Population Structure

A genome-wide scan for selection signatures in two commercial pig breeds was carried out by estimating Fst and CLR at each marker. In the process of quality control, 171121 SNPs and 100 individuals were discards due to missing genotype data, 45600 SNPs and 31825SNPs were removed due to minor allele thresholds and Hardy Weinberg balance thresholds, and 4910 SNPs in sex chromosomes were discarded. After the quality control, 1100 individuals (546 Landrace pigs and 554 Yorkshire pigs) with 92114 SNPs were retained for this analysis (**Tables 1**, **2**). **Table 3** summaries the whole genome potential regions (50 kb) at 18 autonomies by Fst and CLR.

**Table 1:** Information of filtered data and candidate genes detected by Fst.

Method	Items	L-Y
Fst	SNPs	92114
	Outliers	921
	Potential selection regions	540
	Candidate genes	111

**Table 2:** Information of filtered data and candidate genes detected by CLR.

Method	Items	L	Y
CLR	SNPs	92114	92114
	Outliers	921	921
	Potential selection regions	321	628
	Candidate genes	73	125

**Table 3:** Summaries of the numbers of potential selection regions.

Chr	L-Y (Fst)	L (CLR)	Y (CLR)
1	50	49	66
2	36	25	35
3	30	15	42
4	37	16	41
5	23	17	39
6	37	17	39
7	29	21	43
8	43	24	39
9	27	8	51
10	19	16	26
11	19	22	35
12	11	10	10
13	52	19	42
14	42	22	30
15	16	17	27
16	41	9	30
17	16	6	19
18	12	8	14
Total	540	321	628

**FIGURE 1:**
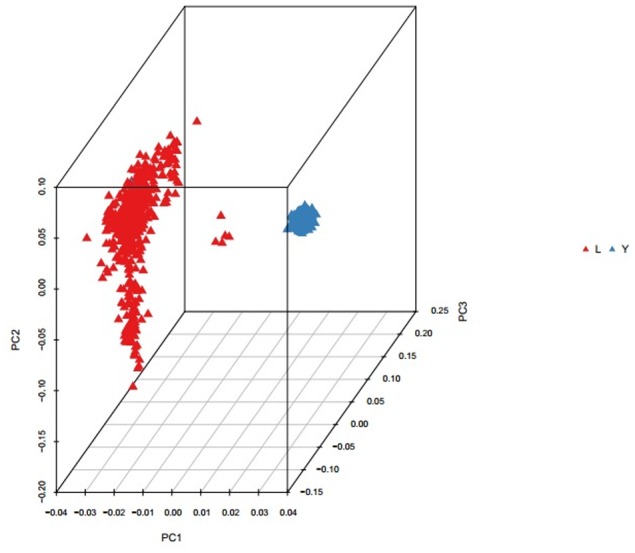
Principal component analysis results based on whole genome SNP data. The red represents the Landrace pigs and the blue represents the Yorkshire pigs.

Population structure was investigated using PCA analysis. The PCA was carried out based on all available SNPs to examine the population genetic structure in this study. As shown in **Figure 1**, most of Landrace pigs and Yorkshire pigs formed a separate cluster severally. Based on the population structure information by PCA, we founded that the raw determination data from the farm had some mistakes about genealogy so we adjusted genealogy for specific individuals in the two breeds.

### Selection Signatures Shared in Two Breeds – Fst Approach

**FIGURE 2:**
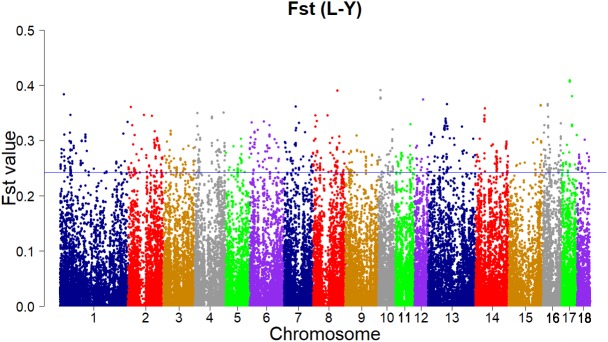
Genome-wide distributions of selection signatures detected by Fst cross all autonomies. The blue line corresponds to the 99% threshold on the corresponding empirical distributions.

According to the empirical distribution of Fst (**Figure 2**), candidate regions under selection were defined as outliers falling with the upper 1% of the distribution of Fst (Fst > 0.243179). Supporting information shows graphically the Fst of each SNP for all 18 autonomies (**Supplementary Figure S1**).

Using this strategy, the result of Fst revealed 111 candidate genes of high levels of differentiation (**Supplementary Table S1**). A gene on chromosome 12 (SSC 12), *ACACA* gene (Fst = 0.373979048), plays a key role in fatty acid biosynthesis (Stachowiak et al., 2013). The acetyl-CoA carboxylase alpha that catalyzes the carboxylation of acetyl-CoA to malonyl-CoA is encoded by the *ACACA* gene. And malonyl-CoA regulates mitochondrial fattyacid β-oxidation and has effects on the expression of hypothalamic neuropeptides modulating energy homeostasis and lipid metabolism (Bionaz and Loor, 2008; Wakil and Abuelheiga, 2009). Some QTLs for average backfat thickness and birth body weight, the percentage of vaccenic, stearic, palmitic, and palmitoleic fatty acids and the total percentage of saturated fatty acids in backfat and/or skeletal muscles are corresponded with the precise localization of *ACACA* gene (Liu et al., 2007, 2008; Quintanilla et al., 2011). On SSC 6, the *MTOR* gene (Fst = 0.33406672) was identified as an important selection signature. *MTOR* gene is related to multiple signaling and signal transduction pathways in various of cellular processes (Hay and Sonenberg, 2004). The *MTOR* gene encodes mammalian target of rapamycin (mTOR), which regulates multiple biological processes such as cellular metabolism, growth and survival in response to hormones, growth factors, nutrients, energy and stress signals as a serine/threonine protein kinase. Moreover, the mTOR signaling pathway regulates many major biological processes and is related to many pathological conditions such as cancer, obesity, type 2 diabetes, and neurodegeneration (Guertin and Sabatini, 2007; Laplante and Sabatini, 2012). An important gene, *GHR* gene (Fst = 0.268147326), which was implicated in promoting the growth and development of animals was deemed as a significant candidate gene on SSC 16 (Leung et al., 1987; Schnoebelen-Combes et al., 1996). The significance of growth hormone (GH) produced by the pituitary gland in supporting growth and development has been known for a long time (Dauncey et al., 1994). The major effect of GH is to promote postnatal longitudinal growth. And GH regulates the lipid, carbohydrate, nitrogen, and mineral metabolism within a cell through interaction with the GH receptor on target cells (Kopchick and Andry, 2000).

### Selection Signatures Unique to Individual Breeds– CLR Approach

**FIGURE 3:**
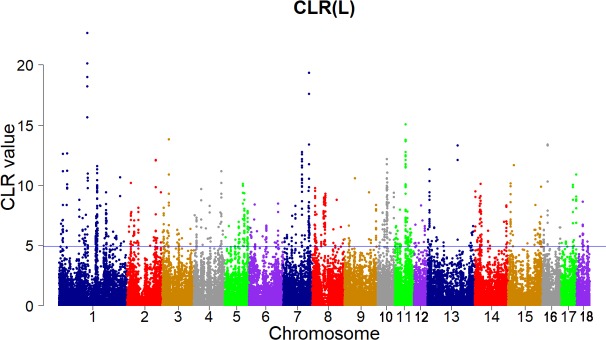
Genome-wide distributions of selection signatures detected by CLR in Landrace cross all autosomes. The blue line corresponds to the 99% threshold on the corresponding empirical distributions.

**FIGURE 4:**
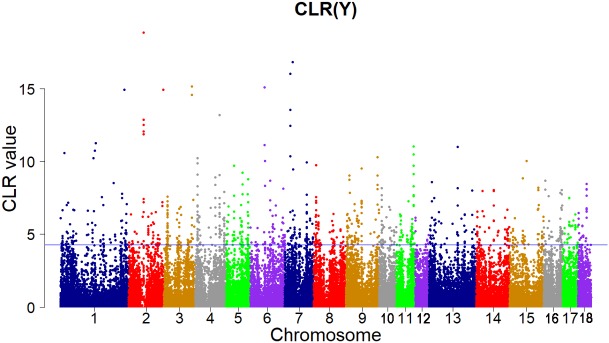
Genome-wide distributions of selection signatures detected by CLR in Yorkshire cross all autosomes. The blue line corresponds to the 99% threshold on the corresponding empirical distributions.

Candidate regions were taken as outliers falling with the 99th percentile of the distribution of CLR (**Figures 3**, **4**). Supporting information shows graphically the CLR of each SNP for all 18 autonomies of Landrace pigs (**Supplementary Figure S1**). Supporting information shows graphically the CLR of each SNP for all 18 autonomies of Yorkshire pigs (**Supplementary Figures S2**, **S3**).

Add up to 73 candidate genes were found for the Landrace pigs using this criterion (CLR > 4.928279, **Supplementary Table S2**). On SSC 4, the *COL11A1* gene (CLR = 11.18224) encodes one of the two alpha chains of type XI collagen which is a minor fibrillar collagen. The type XI collagen in cartilage extracellular matrix is essential for the development of the normal differentiation and spatial organization structure of growth plate chondrocytes (Li et al., 1995). Furthermore, the *COL11A1* gene also shows high evidence of selection in Tibetan wild boar (Li et al., 2013). The *MECR* gene (CLR = 6.377755) on SSC 6 encodes an oxidoreductase that catalyzes the last step in mitochondrial fatty acid synthesis (mtFAS). The mtFAS is a highly conserved metabolic process and is necessary for mitochondrial respiration as a highly conserved metabolic process. It was recently reported that mitochondrial 2-enoyl-CoA/ACP reductase played an important role in placental development in mice (Nair et al., 2017) and the mutations of MECR resulted in childhood-onset dystonia and optic atrophy (Heimer et al., 2016).

A total of 125 candidate genes with high CLR score (CLR > 4.272431) were identified for the Yorkshire pigs based on the distribution of CLR (**Supplementary Table S3**). Some genes play key role in growth, immune and fertility of animals. Two genes on SSC 1, the IGF1R gene (CLR = 6.658113) and the *IGF2R* gene (CLR = 4.433161), were important candidate genes in growth and development of Yorkshire pigs. The insulin-like growth factor (IGF)-system includes insulin-like growth factors 1 and 2 (IGF1 and IGF2) along with the type I (IGF1R) and type II (IGF2R) cell-surface receptors, the insulin receptors (IR) and circulating IGF-binding proteins (IGFBPs) (Wang et al., 2003). IGF1R and IR regulate the biological processes and lead to cell growth, differentiation and survival. In mammals, the activity of IGF2 is mediated by IGF2R, which sequesters IGF2 for internalization and degradation (Brown et al., 2008). The *IFNG* gene (CLR = 6.903143) encodes a soluble cytokine that belongs to the type II interferon (IFN) class. IFNs are cytokines that play a significant role in the resistance of mammalian hosts to pathogens (Boehm et al., 1997). IFN-γ is secreted by thymus-derived (T) cells under specific activation conditions and by natural killer (NK) cells. The biological effects of IFN-γ are regulation of some ways of the immune response, including stimulation of antigen presentation by class I and class II major histocompatibility complex (MHC) molecules, stimulation of bactericidal activity of phagocytes, effects on cell proliferation and apoptosis, orchestration of leukocyte-endothelium interactions (Schroder et al., 2004).

### Functional Annotation of the Candidate Genes

The 111 candidate genes that were identified at high Fst values for Landrace-Yorkshire pair (L-Y), and the 73 and 125 candidate genes that were found by CLR for Landrace pigs and Yorkshire pigs respectively, were accessed for functional enrichment using the DAVID browser. Most of these genes were implicated in multiple signaling and signal transduction pathways in multifarious cellular and biochemical processes (**Supplementary Table S4**). In the cAMP signaling pathway seven candidate genes among them were identified (*CNGB1*, *GABBR1*, *GRIA2*, *HTR4*, *PPARA*, *RAC3*, *TIAM1*). Cyclic adenosine 3′,5′-monophosphate is a member of the most common second messengers. The cAMP is a regulator of pivotal physiologic processes including metabolism, secretion, calcium homeostasis, muscle contraction, cell fate, and gene transcription (Fimia and Sassone-Corsi, 2001). The Jak-STAT signaling pathway contained five candidate genes under selection (*GHR*, *IFNG*, *IL20RB*, *IL23R*, *OSMR*). In mammals, the Jak-STAT pathway is the major signaling mechanism for a wide variety of cytokines and growth factors. Jak-STAT signaling is indispensable for lots of developmental and homeostatic processes, including hematopoiesis, immune cell development, stem cell maintenance, organismal growth, and mammary gland development (Imada and Leonard, 2000; Harrison, 2012; Kiu and Nicholson, 2012). In Wnt signaling pathway, three candidate genes were found (*BAMBI*, *FZD4*, *RAC3*). The Wnt signaling pathway is a regulator in the core biological processes of proliferation, differentiation, stem cell renewal and in the origin of cancer when deregulated (Huelsken and Behrens, 2002). And signaling molecules secreted by the Wnt family have been found to come into play in controlling embryonic development from hydra to human (Yang, 2010). A variety of candidate genes were also identified in pathways upstream and downstream of the mTOR signaling pathway including the insulin signaling pathway (*ACACA*, *FOXO1*, *MTOR*, *PPARA*), the AMPK signaling pathway (*FOXO1*, *IGF1R*), the MAPK signaling pathway (*PTPRR*, *RAC3*), the PI3K-Akt signaling pathway (*COL11A1*, *GHR*, *IGF1R*, *MDM2*, *MTOR*, *OSMR*, *PKN2*, *RAC3*), the glycerolipid metabolism (*AGK*, *DGKH*, *LCLAT1*), the VEGF signaling pathway (*RAC3*), the regulation of autophagy (*ATG7*, *IFNG*) and the regulation of actin cytoskeleton (*PAK5*, *RAC3*, *TIAM1*) (Saltiel and Kahn, 2001; Bevan, 2001; Chen et al., 2001; Cross et al., 2003; Meijer and Codogno, 2004; Engelman et al., 2006; Prentki and Madiraju, 2008; Steinberg and Kemp, 2009; Lee and Dominguez, 2010). Besides, the pathway neuroactive ligand-receptor interaction contained 10 candidate genes (*THRB*, *GRIA2*, *GRIK1*, *GRM7*, *GLRA3*, *HTR4*, *GABBR1*, *NPFFR1*, *PLG*, *GHR*); the metabolic pathways contained 27 candidate genes (*ME1*, *NDST3*, *KYNU*, *HSD17B12*, *ASL*, *STT3B*, *TPK1*, *GALC*, *GALNT16*, *PAFAH1B1*, *RPIA*, *AGK*, *CYP4A24*, *MOCS2*, *MAN1A2*, *BST1*, *ACACA*, *DGKH*, *AK7*, *POLR3C*, *CD38*, *LCLAT1*, *INPP4B*, *GULO*, *OAT*, *MECR*, *PCCB*).

## Discussion

Over the past 300 years, intense artificial selection for production traits has led to the progress of many pig breeds with specialized phenotypic traits. In this study, two different methods, Fst and CLR, were carried out for detecting selection signatures. And then various genes could be deemed as candidate genes based on function or previous study with interesting traits in pig breeds. The same principle of data filtering was adopted to ensure the accuracy of the two methods.

### The Effect of the Methods on the Results

The Fst method is a classical approach for detecting selection signatures based on population differentiation. Under the condition of neutral evolution, the degree of differentiation between populations depends on the genetic drift intensity, as well as the difference in mutation rate and recombination rate in genome. However, when a locus was positively selected on only one population and remained neutral on another, or if the two populations are selected in different directions at same site, the two populations will increase genetic differentiation at that site. Thus, significant differences in allele frequencies between populations can be used as a selective effect of the left blot. On the other hand, if there is a very low degree of differentiation between the two populations in the locus, it may also be caused by a balance selection, a purification selection, or a positive selection in the same direction.

The CLR method uses the combined likelihood of multiple markers to detect the genomic region selected. Besides, the CLR method is an available method for detecting sweeps that is not highly sensitive to assumptions about the underlying recombination rate or recombination hotspots (Williamson et al., 2007; Frantz et al., 2015). Thus, we used CLR test that compared allele frequencies in genome regions to the background pattern of variation so that we can obtain a relatively accurate result. Many other studies indicate that the CLR test has power to detect old selective sweep which occurred in wide time scale. And as we know, domestic pigs originated from wild boar about 9000 years ago so that some old selective sweeps may be detected by CLR test. However, we can not rule out a probability that the CLR test has not detected some old selective sweeps if these genome regions had selected frequently. Even so, we believe that these identified results are very useful to future studies.

### Pig Production Traits

Selection signatures were found that may be involved in some desirable pig production traits. **Table 5** lists the comparison of data on growth traits between two populations.

Traditional pig breeding program have concentrated on growth rate and leanness (Hammond, 1998). However, in order to satisfy the demands of consumers, meat quality has become a an important objective in genetic selection during the last few years (Vidal et al., 2005; Uimari et al., 2013). Fat deposition is a significant trait that directly affects the meat quality. According to the result of Fst, the *ACACA* gene was regarded as a candidate gene that plays an important role in fatty acid biosynthesis. Therefore, genes related to fatty acid biosynthesis were selected by a more intense artificial selection in two breeds. The result of CLR has supported this point: the *MECR* gene detected in Landrace breed was associated with mtFAS. However, no more genes associated with fatty acid biosynthesis were detected by Fst and CLR. The possible explanation is that the two breeds had similar breeding objectives about growth and development for a long time, however, meat quality has not been selected systematically in the last decades. Therefore, most candidate genes were associated with growth and development, and yet a few candidate genes were involved in meat quality.

According to the functional annotation of the candidate genes, several pathways were directly associated with growth and development of animals. For example, the insulin signaling pathway that regulate individual development begin with the binding of insulin to insulin receptors, and thus trigger a series of intracellular signal transduction, ultimately playing a role after reaching the organ (White and Kahn, 1994); the PI3K-Akt signaling pathway is activated by many kinds of cellular stimuli or toxic insults activate the PI3k-Akt signaling pathway which is a regulator for fundamental cellular function including transcription, translation, proliferation, growth and survival (Song et al., 2005). The genes associated with growth and development were identified by Fst and CLR. This suggested that growth and development (growth rate and feed ratio) had been the major breeding goal in the last few decades.

Several genes were identified as candidate genes by both Fst and CLR approaches (**Table 4**). The *IL23R* gene encodes interleukin-23 (IL23) receptor. IL23 is a key factor in innate and adaptive immunity and may participate in acute response to infection in peripheral tissues. IL23 is responsible for autoimmune inflammatory diseases and is important for inflammatory bowel diseases in human (Duerr et al., 2006). Three genes (*DOCK9*, *OAT* and *SPAG17*) were identified by CLR in both Landrace pigs and Yorkshire pigs. The *DOCK9* gene encodes dedicator of cytokinesis protein 9. The *OAT* gene encodes ornithine aminotransferase (OAT) which plays crucial physiological roles in amino acid metabolism (Levillain et al., 2007). The *SPAG17* gene encodes a central pair protein present in the axonemes of cells with a “9 + 2” organization of microtubules. The *SPAG17* gene plays a vital role in the function and structure of motile cilia (Teves et al., 2013).

**Table 4:** Overlapped candidate genes between Fst and CLR.

Genes	SSC	Methods
CNIH3	10	Fst, CLR(Y)
COL14A1	4	Fst, CLR(Y)
DOCK9	11	CLR(L), CLR(Y)
GRM7	13	Fst, CLR(Y)
IL23R	5	Fst, CLR(L)
LRGUK	18	Fst, CLR(Y)
OAT	14	CLR(L), CLR(Y)
SPAG17	4	CLR(L), CLR(Y)
TBC1D5	13	Fst, CLR(Y)

**Table 5:** Comparison of data on growth traits between two populations.

Breeds	Terms	Weight(kg)	BF(mm)	LMT(%)	B100(mm)	D100(d)
Landrace	Mean	107.41	10.10	60.75	9.45	162.64
	Max	136.00	20.30	81.00	20.48	204.20
	Min	75.00	5.00	44.00	4.87	135.90
	*SD*	14.95	2.51	5.94	2.06	11.49
Yorkshire	Mean	107.30	10.18	60.70	9.58	161.08
	Max	159.00	20.00	86.00	15.51	274.45
	Min	75.00	5.00	40.00	4.42	120.59
	*SD*	13.28	2.51	5.56	2.07	11.85

*BF, backfat thickness; LMT, lean muscle thickness; B100, backfat thickness to 100 kg; D100, days to 100 kg; Max, maximum; Min, minimum; SD, standard deviation.*

### Comparison With Previous Studies

Selection signatures detected in this study were compared with previous studies. The *KIT* gene and the *MC1R* gene were important candidate genes relevant to coat color (Andersson and Plastow, 2011; Rubin et al., 2012). The two breeds are all white color, so the two genes were not detected under selection in this study. In addition, the *IGFBP7* gene and the *UNC13C* gene were overlapped with Yang’s report (Yang et al., 2014). Furthermore, a total of 7 genes (*COL11A1*, *COL14A1*, *IFNG*, *IGF2R*, *MTOR*, *MYO10*, *PTPRR*) were overlapped with Li’s study (Li et al., 2013).

## Ethics Statement

All experimental procedures were performed in accordance with the Institutional Review Board (IRB14044) and the Institutional Animal Care and Use Committee of the Sichuan Agricultural University under permit number DKY-B20140302.

## Author Contributions

GT, XL, LZ, YJ, and KW designed the experiments. KW, QY, PW, JZ, and DC performed the data collection. GT, KW, and PW performed the experiments. KW, PW, GT, JM, QT, YJ, AJ, and WX analyzed the data. GT, KW, and PW developed some of the analysis software. KW and GT wrote the manuscript. All authors read and approved the final manuscript.

## Conflict of Interest Statement

The authors declare that the research was conducted in the absence of any commercial or financial relationships that could be construed as a potential conflict of interest.

## Footnotes

1 https://figshare.com/

2 http://www.ensembl.org/

3 http://david.ncifcrf.gov/

4 http://www.genome.jp/kegg/pathway.html

## Supplementary Material

FIGURE S1 Fst value for L-Y breed pair on all autosomes. The red dash line corresponds to the 99% threshold on the corresponding empirical distributions.

FIGURE S2 CLR value for Landrace pigs on all autosomes. The red dash line corresponds to the 99% threshold on the corresponding empirical distributions.

FIGURE S3 CLR value for Yorkshire pigs on all autosomes. The red dash line corresponds to the 99% threshold on the corresponding empirical distributions.

TABLE S1 Summary of candidate genes under selection in high Fst for L-Y breed pair.

TABLE S2 Summary of candidate genes under selection in high CLR in Landrace.

TABLE S3 Summary of candidate genes under selection in high CLR in Yorkshire.

TABLE S4 Enrichment of signaling pathways of the candidate genes by KEGG.
