# Supplementary figures and images for: Detection of Selection Signatures in Chinese Landrace and Yorkshire Pigs Based on Genotyping-by-Sequencing Data
Source: Front Genet. 2018 Apr 9;9:119. doi: 10.3389/fgene.2018.00119 (PMC5900008; doi:10.3389/fgene.2018.00119)

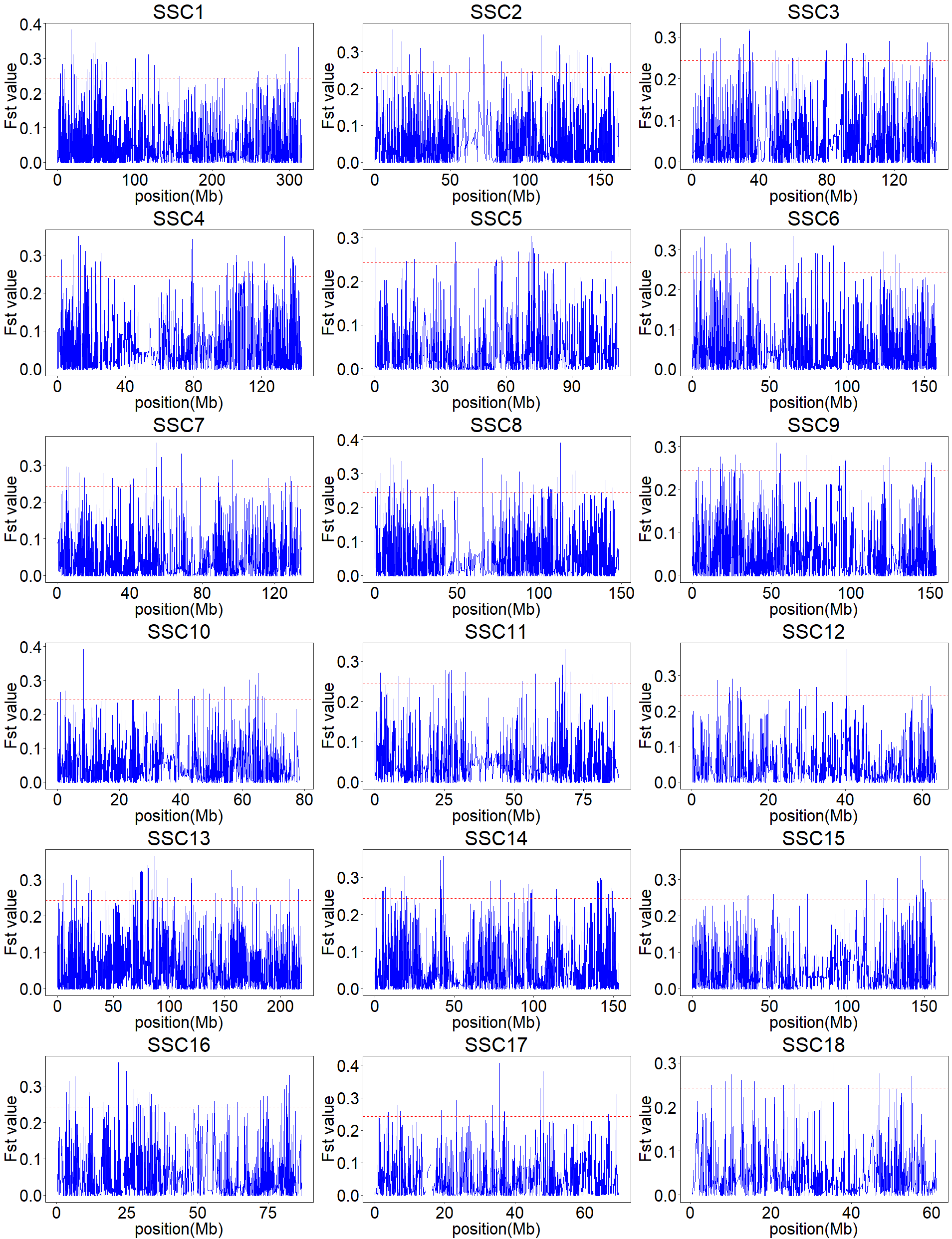

Supplement: FIGURE S1 — Fst value for L-Y breed pair on all autosomes. The red dash line corresponds to the 99% threshold on the corresponding empirical distributions. [file Image_1.TIF]

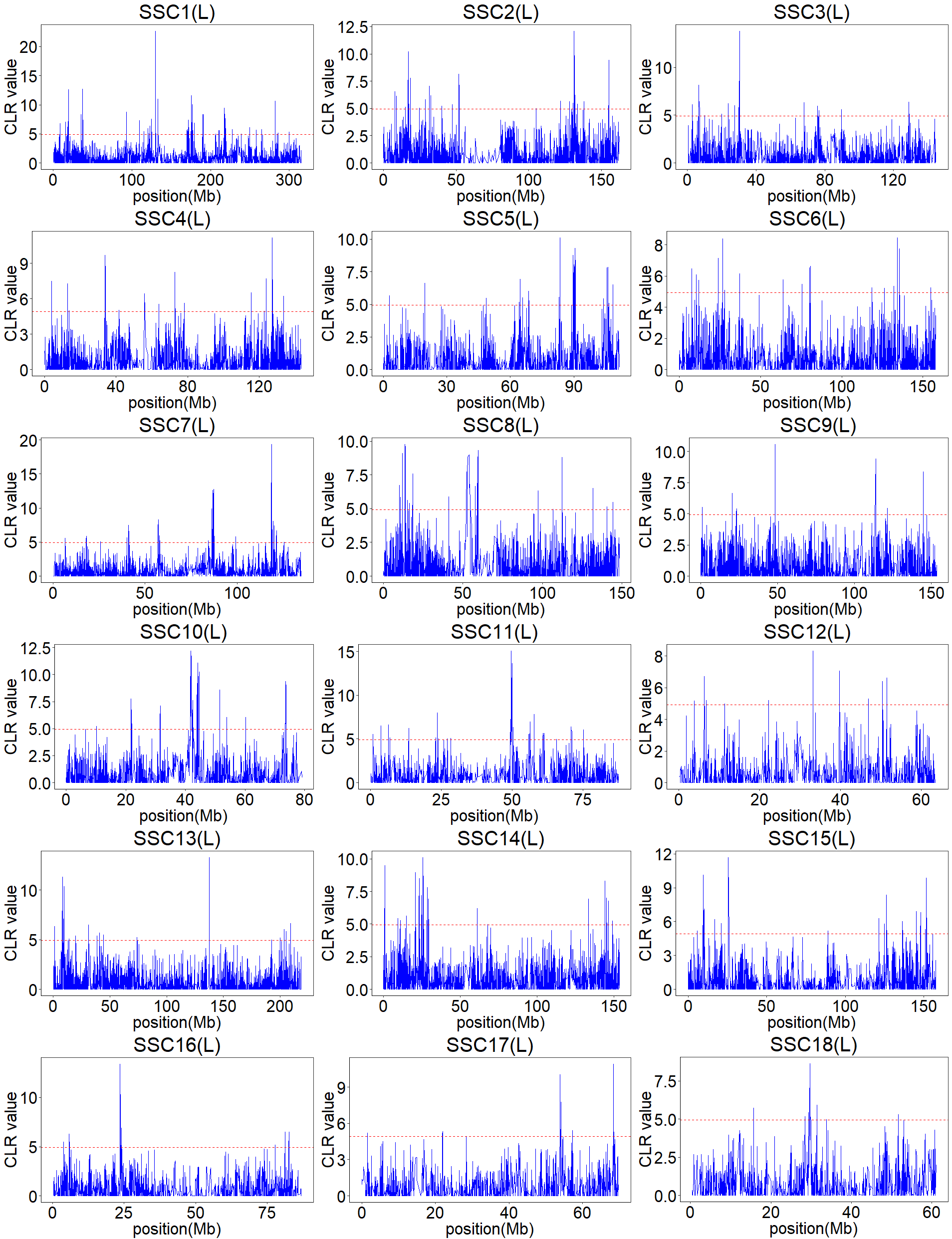

Supplement: FIGURE S2 — CLR value for Landrace pigs on all autosomes. The red dash line corresponds to the 99% threshold on the corresponding empirical distributions. [file Image_2.TIF]

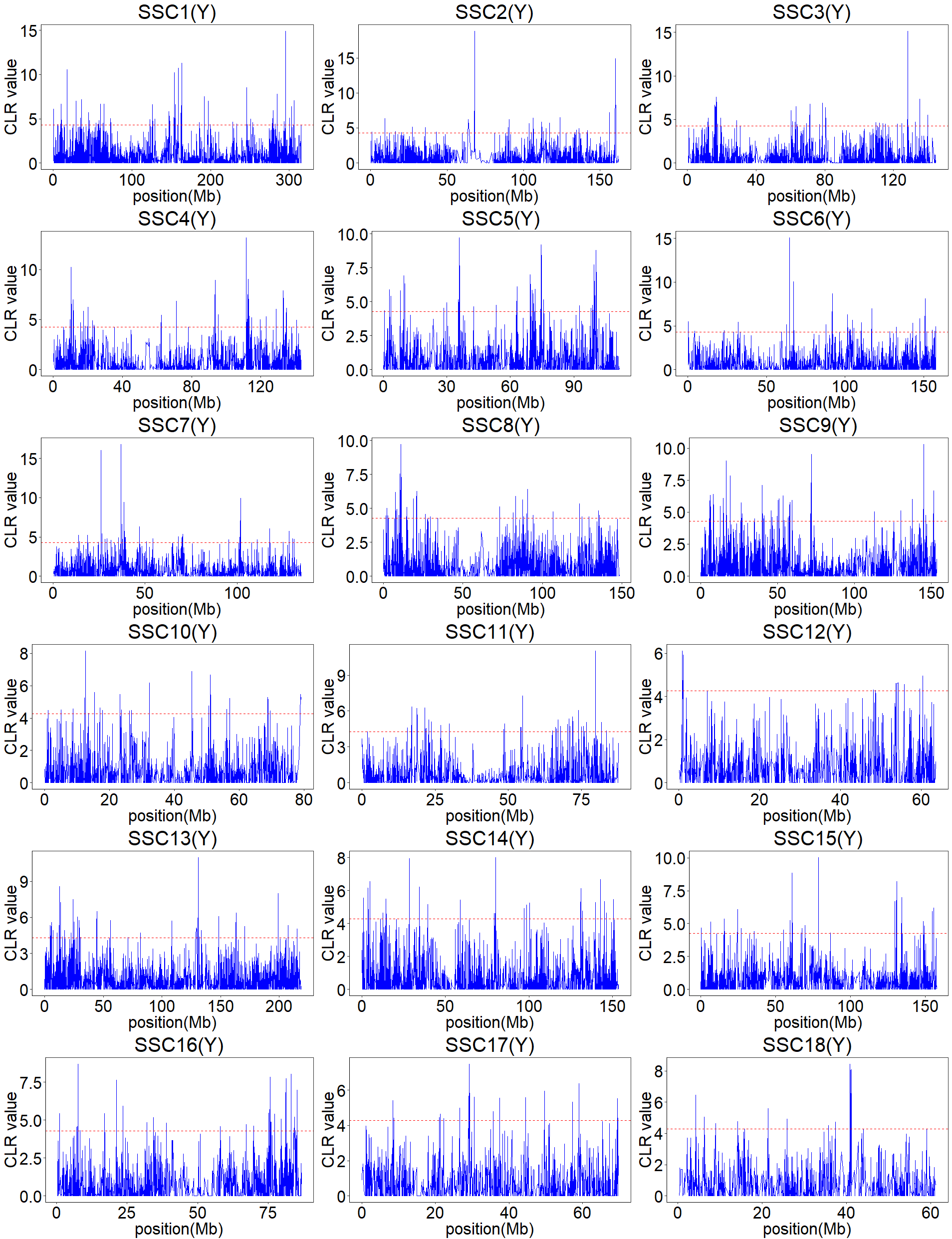

Supplement: FIGURE S3 — CLR value for Yorkshire pigs on all autosomes. The red dash line corresponds to the 99% threshold on the corresponding empirical distributions. [file Image_3.TIF]
