# Supplementary material for: Detection of Selection Signatures in Chinese Landrace and Yorkshire Pigs Based on Genotyping-by-Sequencing Data
Source: Front Genet. 2018 Apr 9;9:119. doi: 10.3389/fgene.2018.00119 (PMC5900008; doi:10.3389/fgene.2018.00119)
Supplement: TABLE S1 — Summary of candidate genes under selection in high Fst for L-Y breed pair. [file Table_1.DOCX]

**Table S1.** **Candidate genes under selection in high Fst for L-Y breed pair.**

| Chr | Position | Gene start (bp) | Gene end (bp) | Fst | Gene stable ID | Within Gene |
| --- | --- | --- | --- | --- | --- | --- |
| 1 | 22131762 | 22050549 | 22136486 | 0.254094453 | ENSSSCG00000004122 | EPM2A |
| 1 | 38228985 | 38212863 | 38839873 | 0.267721998 | ENSSSCG00000004209 | PTPRK |
| 1 | 45805004 | 45615048 | 45819794 | 0.270225622 | ENSSSCG00000004242 | TBC1D32 |
| 1 | 49171210 | 48981862 | 49188743 | 0.283439351 | ENSSSCG00000004250 | SLC35F1 |
| 1 | 49679086 | 49645872 | 49695083 | 0.246021075 | ENSSSCG00000004254 | DCBLD1 |
| 1 | 49862199 | 49755480 | 49862725 | 0.246021075 | ENSSSCG00000026978 | ROS1 |
| 1 | 105351492 | 105323436 | 105382354 | 0.258589895 | ENSSSCG00000004491 | SLC14A2 |
| 1 | 132577534 | 132514137 | 132624890 | 0.243189751 | ENSSSCG00000004621 | MYO5C |
| 1 | 157803929 | 157783503 | 157881006 | 0.248882489 | ENSSSCG00000004832 | UBE3A |
| 1 | 282306629 | 282120743 | 282317913 | 0.254440183 | ENSSSCG00000005455 | SVEP1 |
| 1 | 299885829 | 299771698 | 299984451 | 0.262385864 | ENSSSCG00000005602 | GAPVD1 |
| 2 | 17972275 | 17968507 | 18017928 | 0.327572246 | ENSSSCG00000023974 | PHF21A |
| 2 | 104901808 | 104867963 | 104919387 | 0.245766553 | ENSSSCG00000014163 | SLF1 |
| 2 | 132426666 | 132404679 | 132482857 | 0.245042232 | ENSSSCG00000014240 | CSNK1G3 |
| 2 | 150851170 | 150495712 | 150918983 | 0.256539059 | ENSSSCG00000014399 | ARHGAP26 |
| 2 | 156378326 | 156358304 | 156405576 | 0.266392164 | ENSSSCG00000014427 | FBXO38 |
| 2 | 156477073 | 156451710 | 156556790 | 0.269352561 | ENSSSCG00000014428 | HTR4 |
| 3 | 59844015 | 59834045 | 59864868 | 0.249158366 | ENSSSCG00000008207 | RPIA |
| 3 | 90813072 | 90781750 | 90831103 | 0.256257572 | ENSSSCG00000008402 | CLHC1 |
| 3 | 117066530 | 117059648 | 117102291 | 0.290027988 | ENSSSCG00000008534 | TOGARAM2 |
| 3 | 140635071 | 140570745 | 140645632 | 0.245192989 | ENSSSCG00000028755 | TSSC1 |
| 4 | 19877923 | 19715674 | 19949617 | 0.267790583 | ENSSSCG00000005997 | COL14A1 |
| 4 | 79111362 | 79031984 | 79152558 | 0.342387167 | ENSSSCG00000006231 | CHD7 |
| 4 | 109729936 | 109123026 | 109133240 | 0.256539059 | ENSSSCG00000006691 | POLR3C |
| 4 | 112623846 | 112589262 | 112775639 | 0.252417373 | ENSSSCG00000006726 | SPAG17 |
| 4 | 139825792 | 139804250 | 139885791 | 0.272779136 | ENSSSCG00000006927 | PKN2 |
| 5 | 37424400 | 37376180 | 37645956 | 0.246300709 | ENSSSCG00000025969 | PTPRR |
| 5 | 55275171 | 55270409 | 55298002 | 0.243287343 | ENSSSCG00000000583 | PYROXD1 |
| 5 | 55564959 | 55516474 | 55591616 | 0.247125502 | ENSSSCG00000000585 | SLCO1C1 |
| 5 | 70500306 | 70490239 | 70664042 | 0.265865568 | ENSSSCG00000000755 | ERC1 |
| 5 | 72985212 | 72799795 | 73101239 | 0.264448681 | ENSSSCG00000000778 | CPNE8 |
| 5 | 87105408 | 87094797 | 87321631 | 0.243137571 | ENSSSCG00000000873 | ANO4 |
| 6 | 17840274 | 17785742 | 17839804 | 0.246916843 | ENSSSCG00000002811 | CNGB1 |
| 6 | 42706135 | 42701293 | 42710420 | 0.254460298 | ENSSSCG00000002954 | SPINT2 |
| 6 | 65269960 | 65191722 | 65317849 | 0.33406672 | ENSSSCG00000003413 | MTOR |
| 6 | 77114122 | 77113411 | 77120568 | 0.248194075 | ENSSSCG00000026386 | SLC30A2 |
| 6 | 79840772 | 79677219 | 79876085 | 0.291389228 | ENSSSCG00000003586 | EPB41 |
| 6 | 98537081 | 98533727 | 98538178 | 0.268096445 | ENSSSCG00000003698 | ADCYAP1 |
| 6 | 134270787 | 134219683 | 134283657 | 0.266642412 | ENSSSCG00000003801 | IL23R |
| 7 | 25228312 | 25210913 | 25240202 | 0.278977638 | ENSSSCG00000001242 | GABBR1 |
| 7 | 55062712 | 55050018 | 55108571 | 0.318238407 | ENSSSCG00000001782 | ABHD17C |
| 7 | 116360499 | 116348931 | 116415759 | 0.264344867 | ENSSSCG00000027865 | GALC |
| 7 | 129168324 | 129068962 | 129203801 | 0.256449257 | ENSSSCG00000002526 | RCOR1 |
| 8 | 575665 | 511356 | 626847 | 0.277620228 | ENSSSCG00000008681 | NELFA |
| 8 | 575747 | 527204 | 677341 | 0.275691626 | ENSSSCG00000008684 | POLN |
| 8 | 1434602 | 1370473 | 1444437 | 0.254045711 | ENSSSCG00000008694 | GRK4 |
| 8 | 1514445 | 1461830 | 1586205 | 0.255942137 | ENSSSCG00000008697 | HTT |
| 8 | 11404773 | 11290602 | 11543431 | 0.325291426 | ENSSSCG00000029227 | LDB2 |
| 8 | 15385055 | 15355493 | 15443176 | 0.245995642 | ENSSSCG00000008749 | SLIT2 |
| 8 | 48195245 | 48116708 | 48277325 | 0.247034135 | ENSSSCG00000024685 | GRIA2 |
| 8 | 71454803 | 71421036 | 71477751 | 0.243127848 | ENSSSCG00000025514 | RUFY3 |
| 8 | 76903635 | 76887184 | 76919478 | 0.295178718 | ENSSSCG00000008987 | CCNI |
| 8 | 110793211 | 110753311 | 110850887 | 0.288081784 | ENSSSCG00000009101 | PRDM5 |
| 8 | 112860654 | 112836197 | 112912901 | 0.26345179 | ENSSSCG00000009114 | PRSS12 |
| 8 | 112944858 | 112936929 | 113071813 | 0.389849073 | ENSSSCG00000009115 | NDST3 |
| 8 | 120052109 | 119968858 | 120061196 | 0.295515656 | ENSSSCG00000009132 | ENPEP |
| 8 | 140612644 | 140599657 | 140643507 | 0.246121679 | ENSSSCG00000009222 | SPARCL1 |
| 9 | 124555264 | 124255189 | 124629474 | 0.274333262 | ENSSSCG00000015462 | TPK1 |
| 10 | 15363991 | 15304507 | 15386849 | 0.243575037 | ENSSSCG00000028881 | CNIH3 |
| 10 | 56200852 | 56159320 | 56317981 | 0.243899215 | ENSSSCG00000011075 | KIAA1217 |
| 11 | 1905753 | 1860878 | 1955969 | 0.270483172 | ENSSSCG00000009283 | TNFRSF19 |
| 11 | 25523698 | 25488081 | 25593242 | 0.277052621 | ENSSSCG00000009431 | DGKH |
| 11 | 26374044 | 26327366 | 26385565 | 0.268435503 | ENSSSCG00000009440 | ELF1 |
| 11 | 26440175 | 26394101 | 26459094 | 0.267928099 | ENSSSCG00000009441 | SUGT1 |
| 12 | 40338459 | 40200509 | 40367985 | 0.373979048 | ENSSSCG00000017694 | ACACA |
| 12 | 62227223 | 62221542 | 62261407 | 0.269677472 | ENSSSCG00000021252 | LRRC75A |
| 13 | 4767351 | 4512653 | 4870240 | 0.29146548 | ENSSSCG00000011199 | TBC1D5 |
| 13 | 52494147 | 52485647 | 52650735 | 0.246438728 | ENSSSCG00000011498 | SLC25A26 |
| 13 | 71000304 | 70590017 | 71008682 | 0.261772797 | ENSSSCG00000023891 | GRM7 |
| 13 | 74652325 | 74636984 | 74916092 | 0.320254343 | ENSSSCG00000011575 | ATG7 |
| 13 | 75196117 | 75136046 | 75196974 | 0.324448063 | ENSSSCG00000011578 | TAMM41 |
| 13 | 75376363 | 75371790 | 75377519 | 0.32634481 | ENSSSCG00000029587 | TIMP4 |
| 13 | 84667905 | 84587407 | 84685109 | 0.270062003 | ENSSSCG00000011653 | PCCB |
| 13 | 85578551 | 85560151 | 85581816 | 0.270062003 | ENSSSCG00000025701 | NCK1 |
| 13 | 85578551 | 85592269 | 85626043 | 0.270062003 | ENSSSCG00000022500 | IL20RB |
| 13 | 89202827 | 89149072 | 89299288 | 0.251003214 | ENSSSCG00000011666 | CLSTN2 |
| 13 | 104484379 | 104483992 | 104497533 | 0.251003214 | ENSSSCG00000030567 | SSR3 |
| 13 | 146904320 | 146843104 | 146920228 | 0.248954338 | ENSSSCG00000011870 | PDIA5 |
| 13 | 216190485 | 216148481 | 216190620 | 0.273297572 | ENSSSCG00000029511 | UBASH3A |
| 14 | 6804109 | 6795875 | 6812635 | 0.274046074 | ENSSSCG00000009614 | FAM160B2 |
| 14 | 16765833 | 16704854 | 16784695 | 0.262532872 | ENSSSCG00000009698 | GLRA3 |
| 14 | 41235102 | 41230165 | 41288614 | 0.339698373 | ENSSSCG00000009880 | SLC8B1 |
| 14 | 41333642 | 41316523 | 41380906 | 0.345643778 | ENSSSCG00000009883 | RPH3A |
| 14 | 42255030 | 42204806 | 42293967 | 0.243572053 | ENSSSCG00000009893 | TMEM116 |
| 14 | 79113626 | 79103900 | 79116134 | 0.29277738 | ENSSSCG00000010266 | NPFFR1 |
| 14 | 96419666 | 96392740 | 96440184 | 0.280993059 | ENSSSCG00000010377 | PTPN20 |
| 14 | 96491823 | 96480946 | 96624947 | 0.270427676 | ENSSSCG00000010379 | FRMPD2 |
| 14 | 98059382 | 98025088 | 98075314 | 0.256922095 | ENSSSCG00000010392 | CHAT |
| 14 | 98822634 | 98768454 | 98897870 | 0.266935256 | ENSSSCG00000010403 | MARCH8 |
| 14 | 140747124 | 140637968 | 140875398 | 0.284925295 | ENSSSCG00000010683 | GRK5 |
| 14 | 141865746 | 141831969 | 141983156 | 0.297480747 | ENSSSCG00000010691 | PLPP4 |
| 14 | 143529113 | 143497174 | 143558530 | 0.294174547 | ENSSSCG00000010703 | HTRA1 |
| 14 | 145538004 | 145501611 | 145581693 | 0.255713429 | ENSSSCG00000010732 | FAM53B |
| 14 | 146872798 | 146827125 | 146928694 | 0.25400884 | ENSSSCG00000010745 | FANK1 |
| 14 | 149106813 | 149003756 | 149167170 | 0.27137969 | ENSSSCG00000010755 | PTPRE |
| 15 | 74306325 | 74118964 | 74325810 | 0.259856651 | ENSSSCG00000015885 | PLA2R1 |
| 15 | 144876452 | 144854821 | 144909327 | 0.256731006 | ENSSSCG00000016261 | SP110 |
| 15 | 148688524 | 148620715 | 148702150 | 0.298381873 | ENSSSCG00000016314 | TRPM8 |
| 16 | 4420206 | 4402799 | 4433036 | 0.313786126 | ENSSSCG00000016783 | OTULIN |
| 16 | 6466687 | 6351995 | 6492682 | 0.327280094 | ENSSSCG00000016794 | MYO10 |
| 16 | 24766400 | 24682029 | 24902072 | 0.342446376 | ENSSSCG00000016848 | EGFLAM |
| 16 | 25307255 | 25306092 | 25348992 | 0.24262966 | ENSSSCG00000016851 | OSMR |
| 16 | 28815268 | 28693711 | 28855126 | 0.268147326 | ENSSSCG00000016866 | GHR |
| 16 | 29473282 | 29460089 | 29514351 | 0.255881993 | ENSSSCG00000016873 | NIM1K |
| 16 | 82759753 | 82630444 | 82792519 | 0.330987918 | ENSSSCG00000017109 | ADAMTS16 |
| 17 | 37013298 | 37009320 | 37073251 | 0.257530661 | ENSSSCG00000007166 | PTPRA |
| 17 | 47196478 | 47193722 | 47213176 | 0.328696359 | ENSSSCG00000007349 | ACTR5 |
| 17 | 59425382 | 59377658 | 59514268 | 0.256407738 | ENSSSCG00000007478 | ATP9A |
| 18 | 8658324 | 8555431 | 8689368 | 0.25800484 | ENSSSCG00000016492 | AGK |
| 18 | 15913344 | 15904929 | 16020482 | 0.25770327 | ENSSSCG00000016542 | LRGUK |
| 18 | 25781561 | 25750454 | 25810550 | 0.251043326 | ENSSSCG00000016609 | SLC13A1 |
