# Supplementary material for: Detection of Selection Signatures in Chinese Landrace and Yorkshire Pigs Based on Genotyping-by-Sequencing Data
Source: Front Genet. 2018 Apr 9;9:119. doi: 10.3389/fgene.2018.00119 (PMC5900008; doi:10.3389/fgene.2018.00119)
Supplement: TABLE S2 — Summary of candidate genes under selection in high CLR in Landrace. [file Table_2.DOCX]

**Table S2. Candidate genes under selection in high CLR in Landrace.**

| Chr | Position | Gene start (bp) | Gene end (bp) | CLR | Gene stable ID | Within Gene |
| --- | --- | --- | --- | --- | --- | --- |
| 1 | 8747662 | 8739981 | 8787582 | 6.818471 | ENSSSCG00000004038 | PLG |
| 1 | 18520786 | 18476457 | 18536993 | 6.187937 | ENSSSCG00000004101 | LRP11 |
| 1 | 18809658 | 18783705 | 18819681 | 7.104585 | ENSSSCG00000004109 | ZC3H12D |
| 1 | 93172420 | 93076563 | 93242655 | 8.788745 | ENSSSCG00000004454 | ME1 |
| 1 | 124693436 | 124676343 | 124901532 | 7.535953 | ENSSSCG00000004587 | MYO1E |
| 1 | 125098242 | 124961867 | 125109541 | 5.349079 | ENSSSCG00000004589 | RNF111 |
| 1 | 130063213 | 129783234 | 130430057 | 22.67357 | ENSSSCG00000004614 | UNC13C |
| 1 | 170766448 | 170746448 | 170789634 | 5.253493 | ENSSSCG00000004884 | TMX3 |
| 1 | 176632288 | 176595774 | 176650046 | 9.962848 | ENSSSCG00000004898 | TNFRSF11A |
| 1 | 179121313 | 178905034 | 179129728 | 6.396498 | ENSSSCG00000004907 | CCBE1 |
| 1 | 179236182 | 179212836 | 179239634 | 5.990971 | ENSSSCG00000004909 | CPLX4 |
| 1 | 218637454 | 218624268 | 218670621 | 6.960948 | ENSSSCG00000005126 | PLAA |
| 1 | 218685238 | 218678023 | 218722657 | 7.638105 | ENSSSCG00000005127 | CAAP1 |
| 1 | 264688865 | 264672809 | 264734347 | 5.791932 | ENSSSCG00000005338 | RECK |
| 1 | 285658447 | 285628676 | 285700029 | 5.159856 | ENSSSCG00000005484 | ZNF618 |
| 2 | 7840191 | 7806840 | 7839916 | 6.56073 | ENSSSCG00000021482 | HRASLS5 |
| 2 | 17121918 | 17094654 | 17142309 | 7.128989 | ENSSSCG00000013249 | CKAP5 |
| 2 | 131664612 | 131507534 | 131668175 | 6.229885 | ENSSSCG00000025977 | SNX24 |
| 3 | 6697258 | 6693615 | 6699273 | 7.640846 | ENSSSCG00000007623 | BUD31 |
| 3 | 6701756 | 6699604 | 6713375 | 8.133985 | ENSSSCG00000023257 | PDAP1 |
| 4 | 72847662 | 72746834 | 72921285 | 8.274983 | ENSSSCG00000006199 | PREX2 |
| 4 | 127379842 | 127267390 | 127408467 | 11.18224 | ENSSSCG00000006857 | COL11A1 |
| 5 | 64544366 | 64542869 | 64556408 | 6.942971 | ENSSSCG00000000648 | CLEC7A |
| 5 | 68774312 | 68750885 | 68791407 | 5.281671 | ENSSSCG00000000728 | PARP11 |
| 5 | 83402352 | 83189141 | 83577394 | 10.12155 | ENSSSCG00000000837 | CHST11 |
| 5 | 105609814 | 105477330 | 105610293 | 7.277765 | ENSSSCG00000000938 | LIN7A |
| 5 | 105703416 | 105701192 | 105703369 | 7.815119 | ENSSSCG00000000937 | MYF5 |
| 5 | 105997693 | 105853485 | 106003580 | 5.281197 | ENSSSCG00000000943 | OTOGL |
| 6 | 80009066 | 79984219 | 80018092 | 6.377755 | ENSSSCG00000003589 | MECR |
| 6 | 134268848 | 134219683 | 134283657 | 8.464533 | ENSSSCG00000003801 | IL23R |
| 6 | 135445558 | 135397188 | 135560153 | 7.759777 | ENSSSCG00000003807 | DNAJC6 |
| 7 | 57086762 | 57790943 | 57867976 | 8.285484 | ENSSSCG00000001808 | CPEB1 |
| 7 | 57985901 | 57896450 | 58089348 | 5.199719 | ENSSSCG00000001810 | PDE8A |
| 7 | 99206894 | 99153545 | 99263645 | 5.816966 | ENSSSCG00000002306 | GALNT16 |
| 7 | 118530084 | 118431048 | 118546572 | 19.37196 | ENSSSCG00000002432 | KCNK13 |
| 7 | 119430538 | 119404956 | 119444055 | 8.083755 | ENSSSCG00000002438 | C14orf159 |
| 7 | 119555012 | 119490916 | 119618608 | 7.411862 | ENSSSCG00000002440 | CCDC88C |
| 7 | 125295606 | 125267394 | 125310472 | 4.988774 | ENSSSCG00000002506 | VRK1 |
| 8 | 10732701 | 10731289 | 10781193 | 5.858607 | ENSSSCG00000008742 | CD38 |
| 8 | 41176379 | 41159617 | 41236217 | 5.884177 | ENSSSCG00000008831 | DCUN1D4 |
| 8 | 59109829 | 59064675 | 59139438 | 6.888611 | ENSSSCG00000008913 | IGFBP7 |
| 8 | 112534366 | 112415522 | 112556423 | 8.806443 | ENSSSCG00000009111 | SYNPO2 |
| 8 | 144303368 | 144298649 | 144314413 | 5.473114 | ENSSSCG00000009234 | FAM175A |
| 9 | 23005163 | 22997809 | 23006976 | 5.19707 | ENSSSCG00000014920 | FZD4 |
| 9 | 114125181 | 114070908 | 114333182 | 5.51135 | ENSSSCG00000015426 | RELN |
| 9 | 144808809 | 144806836 | 144936784 | 7.005048 | ENSSSCG00000021068 | TRAF5 |
| 10 | 44170722 | 44120645 | 44210868 | 11.06329 | ENSSSCG00000011013 | WAC |
| 10 | 44243709 | 44241604 | 44248652 | 9.836719 | ENSSSCG00000011014 | BAMBI |
| 11 | 13431898 | 13275420 | 13486078 | 6.218967 | ENSSSCG00000009362 | TRPC4 |
| 11 | 49593352 | 49565676 | 49594964 | 13.76762 | ENSSSCG00000025120 | BORA |
| 11 | 49593419 | 49590875 | 49614012 | 15.07568 | ENSSSCG00000009460 | DIS3 |
| 11 | 56159975 | 56098099 | 56173051 | 5.602368 | ENSSSCG00000009479 | RBM26 |
| 11 | 75158934 | 74997784 | 75158789 | 5.516511 | ENSSSCG00000022112 | DOCK9 |
| 12 | 22277666 | 22275243 | 22290067 | 5.191801 | ENSSSCG00000017472 | IGFBP4 |
| 12 | 47025076 | 47004047 | 47033187 | 5.292347 | ENSSSCG00000017774 | FAM222B |
| 12 | 50449330 | 50415347 | 50501646 | 6.417722 | ENSSSCG00000017836 | PAFAH1B1 |
| 13 | 19718434 | 19678183 | 19778925 | 5.36823 | ENSSSCG00000011229 | STT3B |
| 13 | 38519148 | 38462228 | 38525371 | 5.374632 | ENSSSCG00000022616 | DCP1A |
| 13 | 137336043 | 137315798 | 137369721 | 13.29856 | ENSSSCG00000021206 | IL1RAP |
| 13 | 203535021 | 203411536 | 203632118 | 6.051901 | ENSSSCG00000025119 | GRIK1 |
| 13 | 205253114 | 205172409 | 205307822 | 5.859412 | ENSSSCG00000012034 | TIAM1 |
| 14 | 24754825 | 24748336 | 24847567 | 7.213225 | ENSSSCG00000028018 | EP400 |
| 14 | 25759250 | 25517652 | 25788741 | 8.460695 | ENSSSCG00000009745 | ADGRD1 |
| 14 | 29391780 | 29391629 | 29617748 | 6.907953 | ENSSSCG00000009754 | TMEM132B |
| 14 | 145296410 | 145293480 | 145296034 | 7.002067 | ENSSSCG00000010733 | NKX1-2 |
| 14 | 146351402 | 145318780 | 145341081 | 6.789529 | ENSSSCG00000010734 | OAT |
| 15 | 9619006 | 9609578 | 9821863 | 10.13811 | ENSSSCG00000015673 | KYNU |
| 15 | 121328203 | 121306038 | 121474753 | 6.300355 | ENSSSCG00000016131 | ADAM23 |
| 15 | 124739501 | 124563202 | 124740175 | 5.809642 | ENSSSCG00000022830 | KANSL1L |
| 15 | 144959644 | 144908676 | 144973706 | 6.91547 | ENSSSCG00000016262 | SP140 |
| 16 | 23782060 | 23761201 | 24139970 | 10.29987 | ENSSSCG00000016846 | WDR70 |
| 16 | 77790934 | 77761983 | 77799297 | 5.171718 | ENSSSCG00000023512 | SLC36A1 |
| 17 | 54156699 | 54155730 | 54168471 | 7.768909 | ENSSSCG00000022777 | SLC35C2 |
