# Supplementary material for: Detection of Selection Signatures in Chinese Landrace and Yorkshire Pigs Based on Genotyping-by-Sequencing Data
Source: Front Genet. 2018 Apr 9;9:119. doi: 10.3389/fgene.2018.00119 (PMC5900008; doi:10.3389/fgene.2018.00119)
Supplement: TABLE S3 — Summary of candidate genes under selection in high CLR in Yorkshire. [file Table_3.DOCX]

**Table S3. Candidate genes under selection in high CLR in Yorkshire.**

| Chr | Position | Gene start (bp) | Gene end (bp) | CLR | Gene stable ID | Within Gene |
| --- | --- | --- | --- | --- | --- | --- |
| 1 | 9220090 | 9166935 | 9271240 | 4.433161 | ENSSSCG00000004044 | IGF2R |
| 1 | 10490198 | 10432694 | 10496126 | 4.865943 | ENSSSCG00000004057 | SYTL3 |
| 1 | 34440267 | 34439083 | 34441686 | 5.072898 | ENSSSCG00000004177 | RPS12 |
| 1 | 34440267 | 64482243 | 64542250 | 5.072898 | ENSSSCG00000004322 | ANKRD6 |
| 1 | 57316395 | 123140555 | 123240772 | 4.332197 | ENSSSCG00000004576 | RORA |
| 1 | 64511059 | 153514790 | 153853782 | 6.658113 | ENSSSCG00000004812 | IGF1R |
| 1 | 123149206 | 302917898 | 302944879 | 4.939087 | ENSSSCG00000005652 | GLE1 |
| 1 | 153642906 | 305502184 | 305633144 | 10.2117 | ENSSSCG00000005719 | RAPGEF1 |
| 1 | 302922273 | 34439763 | 34439839 | 6.385128 | ENSSSCG00000018450 | SNORD100 |
| 1 | 305617411 | 57276096 | 57378530 | 4.285235 | ENSSSCG00000026246 | B3GAT2 |
| 2 | 20077722 | 27175381 | 27490459 | 4.366566 | ENSSSCG00000013294 | LDLRAD3 |
| 2 | 27438022 | 89844360 | 89996126 | 5.152543 | ENSSSCG00000014113 | HOMER1 |
| 2 | 89864128 | 112016541 | 112091668 | 5.099474 | ENSSSCG00000014183 | SLCO6A1 |
| 2 | 112058679 | 20061566 | 20211950 | 5.122444 | ENSSSCG00000021739 | HSD17B12 |
| 3 | 16899824 | 16890467 | 16899578 | 6.927998 | ENSSSCG00000007738 | ASL |
| 3 | 19333667 | 109677473 | 109691729 | 5.152088 | ENSSSCG00000008497 | GPATCH11 |
| 3 | 109692202 | 109692688 | 109810357 | 4.625819 | ENSSSCG00000008498 | HEATR5B |
| 3 | 109692202 | 122136104 | 122272144 | 4.625819 | ENSSSCG00000008593 | KLHL29 |
| 3 | 115412400 | 128326292 | 128396035 | 4.460148 | ENSSSCG00000008615 | VSNL1 |
| 3 | 122136755 | 19136264 | 19387999 | 4.458281 | ENSSSCG00000022785 | GSG1L |
| 3 | 128336627 | 115371533 | 115541170 | 15.14778 | ENSSSCG00000025901 | LCLAT1 |
| 4 | 15926894 | 15725526 | 15935437 | 4.679519 | ENSSSCG00000005977 | TMEM65 |
| 4 | 18468749 | 18459354 | 18488950 | 4.344963 | ENSSSCG00000005992 | SHAS2 |
| 4 | 19802126 | 19715674 | 19949617 | 4.299473 | ENSSSCG00000005997 | COL14A1 |
| 4 | 20023578 | 19989287 | 20171545 | 6.245 | ENSSSCG00000005998 | DEPTOR |
| 4 | 112001892 | 111992595 | 112035055 | 5.034204 | ENSSSCG00000006725 | TBX15 |
| 4 | 112767556 | 112589262 | 112775639 | 9.066169 | ENSSSCG00000006726 | SPAG17 |
| 4 | 113323687 | 113184591 | 113342908 | 5.359709 | ENSSSCG00000006730 | MAN1A2 |
| 4 | 114763215 | 114752826 | 114845145 | 5.179615 | ENSSSCG00000006743 | SLC22A15 |
| 4 | 128868010 | 128863165 | 128939924 | 6.040978 | ENSSSCG00000006860 | SLC30A7 |
| 4 | 134950660 | 134846031 | 134978491 | 4.703805 | ENSSSCG00000006890 | ABCA4 |
| 5 | 476260 | 424929 | 488440 | 4.354083 | ENSSSCG00000000006 | PPARA |
| 5 | 8153446 | 9688037 | 9742182 | 5.830244 | ENSSSCG00000000152 | RBFOX2 |
| 5 | 9710608 | 35374323 | 35379577 | 6.903143 | ENSSSCG00000000481 | IFNG |
| 5 | 35376254 | 35889682 | 36061995 | 5.16055 | ENSSSCG00000000488 | MDM2 |
| 5 | 35861278 | 69417021 | 69481222 | 9.717738 | ENSSSCG00000000736 | TEAD4 |
| 5 | 63081427 | 74510800 | 74656333 | 5.029732 | ENSSSCG00000000784 | LRRK2 |
| 5 | 69458124 | 8149172 | 8167615 | 7.007379 | ENSSSCG00000021944 | RAC3 |
| 5 | 74565787 | 63079116 | 63326668 | 9.212528 | ENSSSCG00000024152 | ETV6 |
| 6 | 4178716 | 4161680 | 4206391 | 4.426432 | ENSSSCG00000002666 | KIAA0513 |
| 6 | 64650063 | 91860921 | 91907112 | 11.12173 | ENSSSCG00000003680 | RALBP1 |
| 6 | 91907379 | 101523030 | 101634463 | 8.687514 | ENSSSCG00000003710 | TTC39C |
| 6 | 101560248 | 104448291 | 104558275 | 6.277391 | ENSSSCG00000003721 | CHST9 |
| 6 | 104503101 | 105223854 | 105518711 | 5.68395 | ENSSSCG00000003722 | CDH2 |
| 6 | 105439089 | 142972837 | 143127238 | 4.71482 | ENSSSCG00000003833 | DAB1 |
| 6 | 143113442 | 147320008 | 147467617 | 5.302791 | ENSSSCG00000003859 | ZCCHC11 |
| 6 | 147361292 | 157479913 | 157721955 | 4.461015 | ENSSSCG00000003983 | SMAP2 |
| 6 | 151960460 | 155424281 | 155485848 | 4.324048 | ENSSSCG00000023904 | CFAP57 |
| 6 | 151960460 | 151954326 | 152007678 | 4.324048 | ENSSSCG00000024129 | CYP4X1 |
| 6 | 155468307 | 151929629 | 152153725 | 4.541618 | ENSSSCG00000024778 | CYP4A24 |
| 6 | 157644150 | 64649453 | 64650803 | 4.93031 | ENSSSCG00000027822 | CORT |
| 7 | 37389856 | 37314126 | 37411217 | 6.612148 | ENSSSCG00000001567 | CPNE5 |
| 7 | 39306182 | 39286129 | 39569290 | 4.938329 | ENSSSCG00000001588 | DNAH8 |
| 7 | 95304508 | 95193213 | 95335426 | 4.666963 | ENSSSCG00000002277 | SPTB |
| 7 | 117897610 | 117530994 | 117907020 | 4.293756 | ENSSSCG00000002429 | FOXN3 |
| 7 | 124920376 | 124867919 | 124925154 | 4.353831 | ENSSSCG00000002504 | AK7 |
| 7 | 127995553 | 127973226 | 128046488 | 5.717838 | ENSSSCG00000002508 | SETD3 |
| 7 | 129976171 | 129852900 | 129998766 | 4.788392 | ENSSSCG00000002540 | PPP2R5C |
| 8 | 10505916 | 10399765 | 10545919 | 4.542517 | ENSSSCG00000008738 | CC2D2A |
| 8 | 10598797 | 10562901 | 10609521 | 6.335013 | ENSSSCG00000008740 | FBXL5 |
| 8 | 10675054 | 87282708 | 87651904 | 5.427048 | ENSSSCG00000009035 | SLC10A7 |
| 8 | 87572965 | 90608437 | 90811111 | 5.627826 | ENSSSCG00000009050 | INPP4B |
| 8 | 90685014 | 10659177 | 10694532 | 6.400629 | ENSSSCG00000028359 | BST1 |
| 9 | 8165153 | 8130490 | 8362269 | 6.385949 | ENSSSCG00000014820 | FCHSD2 |
| 9 | 14538371 | 14329065 | 14562328 | 6.095424 | ENSSSCG00000014894 | TENM4 |
| 9 | 25981165 | 35088725 | 35526955 | 4.330523 | ENSSSCG00000014975 | CNTN5 |
| 9 | 35163808 | 41043564 | 41065077 | 4.487832 | ENSSSCG00000015009 | KDELC2 |
| 9 | 40034149 | 41073546 | 41217329 | 7.079766 | ENSSSCG00000015010 | EXPH5 |
| 9 | 41049647 | 46331476 | 46367854 | 4.492126 | ENSSSCG00000015053 | HTR3B |
| 9 | 41177082 | 50441613 | 50474695 | 4.736786 | ENSSSCG00000015086 | TMPRSS4 |
| 9 | 46361874 | 57661819 | 57731502 | 4.605936 | ENSSSCG00000015206 | CCDC15 |
| 9 | 50441982 | 130015440 | 130283638 | 5.919005 | ENSSSCG00000015512 | PAPPA2 |
| 9 | 57669615 | 146503649 | 146520800 | 5.571022 | ENSSSCG00000015619 | CAMK1G |
| 9 | 130263732 | 40006179 | 40106982 | 5.126679 | ENSSSCG00000028201 | CWF19L2 |
| 9 | 146512554 | 25981442 | 25994185 | 4.776934 | ENSSSCG00000030953 | CH242-134A11.1 |
| 10 | 8717802 | 8644061 | 8823152 | 4.584088 | ENSSSCG00000010814 | ESRRG |
| 10 | 13582755 | 13487025 | 13583457 | 4.298339 | ENSSSCG00000010834 | MIA3 |
| 10 | 15364212 | 16930456 | 17170362 | 5.604421 | ENSSSCG00000010862 | CDC42BPA |
| 10 | 16980914 | 51148107 | 51300010 | 4.63208 | ENSSSCG00000011047 | FAM171A1 |
| 10 | 51245657 | 15304507 | 15386849 | 5.449242 | ENSSSCG00000028881 | CNIH3 |
| 11 | 15557963 | 15544860 | 15688360 | 4.584008 | ENSSSCG00000009370 | FOXO1 |
| 11 | 18884914 | 22822397 | 22984001 | 5.686451 | ENSSSCG00000009422 | TSC22D1 |
| 11 | 22855309 | 23611635 | 23650607 | 5.269626 | ENSSSCG00000009426 | CCDC122 |
| 11 | 23645503 | 72009864 | 72063632 | 5.117665 | ENSSSCG00000009501 | DZIP1 |
| 11 | 72061777 | 74997784 | 75158789 | 5.507681 | ENSSSCG00000022112 | DOCK9 |
| 11 | 72123930 | 18866090 | 18916064 | 5.141705 | ENSSSCG00000022623 | CDADC1 |
| 11 | 75005902 | 72092330 | 72161779 | 4.693267 | ENSSSCG00000026082 | DNAJC3 |
| 12 | 1114724 | 54332090 | 54338827 | 5.927009 | ENSSSCG00000017916 | TM4SF5 |
| 12 | 54336029 | 1102655 | 1126695 | 4.62853 | ENSSSCG00000024235 | NPLOC4 |
| 13 | 4769065 | 4512653 | 4870240 | 5.329814 | ENSSSCG00000011199 | TBC1D5 |
| 13 | 12441749 | 12399955 | 12493717 | 8.593797 | ENSSSCG00000011211 | THRB |
| 13 | 29365562 | 29344740 | 29398346 | 5.755553 | ENSSSCG00000011295 | SNRK |
| 13 | 29763450 | 29629877 | 29791476 | 5.130364 | ENSSSCG00000011296 | ANO10 |
| 13 | 44227045 | 131074737 | 131180554 | 5.891502 | ENSSSCG00000011776 | YEATS2 |
| 13 | 70977390 | 148398446 | 148474820 | 4.326284 | ENSSSCG00000011882 | GOLGB1 |
| 13 | 131124303 | 162987676 | 163247801 | 8.167023 | ENSSSCG00000011946 | ALCAM |
| 13 | 148409493 | 44223067 | 44230736 | 6.065967 | ENSSSCG00000022131 | KCTD6 |
| 13 | 163233619 | 70590017 | 71008682 | 5.156015 | ENSSSCG00000023891 | GRM7 |
| 14 | 12493799 | 12480095 | 12512560 | 4.407114 | ENSSSCG00000009667 | GULO |
| 14 | 12530072 | 12516232 | 12531189 | 4.644541 | ENSSSCG00000009668 | CLU |
| 14 | 58795875 | 58794722 | 58843156 | 5.419445 | ENSSSCG00000010145 | HEATR1 |
| 14 | 58864860 | 58841260 | 58871583 | 5.448711 | ENSSSCG00000010146 | LGALS8 |
| 14 | 79183174 | 79134988 | 79214217 | 4.622271 | ENSSSCG00000010267 | LRRC20 |
| 14 | 80125321 | 80113947 | 80157534 | 5.952011 | ENSSSCG00000010277 | SLC29A3 |
| 14 | 97240832 | 97166335 | 97427141 | 4.913752 | ENSSSCG00000010383 | WDFY4 |
| 14 | 131318303 | 131288632 | 131418839 | 4.700259 | ENSSSCG00000010621 | ADD3 |
| 14 | 132097667 | 132090688 | 132146151 | 4.331886 | ENSSSCG00000010626 | RBM20 |
| 14 | 142824205 | 142698096 | 142860718 | 6.692321 | ENSSSCG00000010699 | ATE1 |
| 14 | 145323918 | 145318780 | 145341081 | 5.348297 | ENSSSCG00000010734 | OAT |
| 14 | 145407773 | 145381300 | 145499300 | 5.070917 | ENSSSCG00000010735 | LHPP |
| 14 | 150738218 | 150607123 | 150887541 | 5.47863 | ENSSSCG00000010756 | MGMT |
| 15 | 26938792 | 26872992 | 26974155 | 4.633264 | ENSSSCG00000015714 | CCDC93 |
| 15 | 55369448 | 55338007 | 55406429 | 4.520835 | ENSSSCG00000015820 | NSD3 |
| 15 | 66975011 | 66699447 | 67026722 | 4.290404 | ENSSSCG00000015866 | FMNL2 |
| 15 | 134265988 | 134233624 | 134318259 | 7.009052 | ENSSSCG00000016213 | GLB1L |
| 15 | 148551775 | 148541192 | 148557762 | 5.172157 | ENSSSCG00000016313 | HJURP |
| 16 | 21219580 | 21107618 | 21337349 | 7.619723 | ENSSSCG00000016824 | RAI14 |
| 16 | 23507833 | 23393877 | 23511836 | 5.916973 | ENSSSCG00000016842 | NIPBL |
| 16 | 32023866 | 31878944 | 32060032 | 4.824114 | ENSSSCG00000016882 | PARP8 |
| 16 | 34269304 | 34269654 | 34290773 | 5.160793 | ENSSSCG00000016888 | MOCS2 |
| 17 | 20988302 | 20830938 | 21009099 | 4.437671 | ENSSSCG00000007061 | PAK5 |
| 17 | 29170804 | 29095048 | 29329854 | 7.488378 | ENSSSCG00000007083 | PCSK2 |
| 18 | 15924137 | 15904929 | 16020482 | 4.277834 | ENSSSCG00000016542 | LRGUK |
| 18 | 40957838 | 41062876 | 41246184 | 8.058183 | ENSSSCG00000016657 | AOAH |
| 18 | 41193527 | 40957727 | 40957845 | 8.090847 | ENSSSCG00000018656 | 5S_rRNA |
