# Supplementary material for: Detection of Selection Signatures in Chinese Landrace and Yorkshire Pigs Based on Genotyping-by-Sequencing Data
Source: Front Genet. 2018 Apr 9;9:119. doi: 10.3389/fgene.2018.00119 (PMC5900008; doi:10.3389/fgene.2018.00119)
Supplement: TABLE S4 — Enrichment of signaling pathways of the candidate genes by KEGG. [file Table_4.DOCX]

**Table S4.** **Enrichment of signaling pathways of the candidate genes by KEGG.**

| Database | Term | Genes |
| --- | --- | --- |
| KEGG | ssc04520 Adherens junction | IGF1R, RAC3 |
| KEGG | ssc04920 Adipocytokine signaling pathway | MTOR, PPARA |
| KEGG | ssc05146 Amoebiasis | COL11A1, IFNG |
| KEGG | ssc04152 AMPK signaling pathway | ACACA, FOXO1, IGF1R, MTOR |
| KEGG | ssc05014 Amyotrophic lateral sclerosis (ALS) | GRIA2, RAC3 |
| KEGG | ssc04360 Axon guidance | PAK5, RAC3, SLIT2 |
| KEGG | ssc01230 Biosynthesis of amino acids | ASL, RPIA |
| KEGG | ssc01130 Biosynthesis of antibiotics | AK7, ASL, OAT, PCCB, RPIA |
| KEGG | ssc04020 Calcium signaling pathway | CD38, HTR4 |
| KEGG | ssc04024 cAMP signaling pathway | CNGB1, GABBR1, GRIA2, HTR4, PPARA, RAC3, TIAM1 |
| KEGG | ssc01200 Carbon metabolism | ME1, PCCB, RPIA |
| KEGG | ssc04062 Chemokine signaling pathway | GRK4, GRK5, RAC3, TIAM1 |
| KEGG | ssc05231 Choline metabolism in cancer | DGKH, MTOR, RAC3 |
| KEGG | ssc04060 Cytokine-cytokine receptor interaction | GHR, IFNG, IL1RAP, IL20RB, IL23R, OSMR, TNFRSF11A, TNFRSF19 |
| KEGG | ssc04320 Dorso-ventral axis formation | CPEB1, ETV6 |
| KEGG | ssc04144 Endocytosis | DNAJC6, GRK4, GRK5, IGF1R, IGF2R, MDM2, SMAP2 |
| KEGG | ssc05169 Epstein-Barr virus infection | CD38, IFNG, MDM2, POLR3C |
| KEGG | ssc04012 ErbB signaling pathway | MTOR, NCK1, PAK5 |
| KEGG | ssc04910 Insulin signaling pathway | ACACA, FOXO1, MTOR, RAPGEF1 |
| KEGG | ssc04630 Jak-STAT signaling pathway | GHR, IFNG, IL20RB, IL23R, IL23R, OSMR |
| KEGG | ssc04730 Long-term depression | GRIA2, IGF1R |
| KEGG | ssc04142 Lysosome | GALC, IGF2R |
| KEGG | ssc04010 MAPK signaling pathway | PTPRR, RAC3 |
| KEGG | ssc05218 Melanoma | IGF1R, MDM2 |
| KEGG | ssc01100 Metabolic pathways | ACACA, AGK, AK7, ASL, BST1, CD38, CYP4A24, DGKH, GALC, GALNT16, GULO, HSD17B12, INPP4B, KYNU, LCLAT1, MAN1A2, ME1, MECR, MOCS2, NDST3, OAT, OAT, PAFAH1B1, PCCB, POLR3C, RPIA, STT3B, TPK1 |
| KEGG | ssc05206 MicroRNAs in cancer | MDM2, MTOR, RECK |
| KEGG | ssc05032 Morphine addiction | GABBR1, GRK4, GRK5, PDE8A |
| KEGG | ssc04650 Natural killer cell mediated cytotoxicity | IFNG, RAC3 |
| KEGG | ssc04080 Neuroactive ligand-receptor interaction | GABBR1, GHR, GLRA3, GRIA2, GRIK1, GRM7, HTR4, NPFFR1, PLG, THRB |
| KEGG | ssc04722 Neurotrophin signaling pathway | RAC3, RAPGEF1 |
| KEGG | ssc04064 NF-kappa B signaling pathway | ERC1, TNFRSF11A |
| KEGG | ssc00510 N-Glycan biosynthesis | MAN1A2, STT3B |
| KEGG | ssc00760 Nicotinate and nicotinamide metabolism | BST1, CD38 |
| KEGG | ssc04932 Non-alcoholic fatty liver disease (NAFLD) | PPARA, RAC3 |
| KEGG | ssc04114 Oocyte meiosis | CPEB1, IGF1R |
| KEGG | ssc04380 Osteoclast differentiation | IFNG, RAC3, TNFRSF11A |
| KEGG | ssc04921 Oxytocin signaling pathway | CAMK1G, CD38 |
| KEGG | ssc05212 Pancreatic cancer | RAC3, RALBP1 |
| KEGG | ssc04972 Pancreatic secretion | BST1, CD38, RAC3, |
| KEGG | ssc05200 Pathways in cancer | FOXO1, FZD4, IGF1R, MDM2, MTOR, RAC3, RALBP1 |
| KEGG | ssc04145 Phagosome | CLEC7A, PLA2R1, RAC3 |
| KEGG | ssc04070 Phosphatidylinositol signaling system | DGKH, INPP4B |
| KEGG | ssc04151 PI3K-Akt signaling pathway | COL11A1, GHR, IGF1R, MDM2, MTOR, OSMR, PKN2, RAC3 |
| KEGG | ssc03320 PPAR signaling pathway | CYP4A24, PPARA |
| KEGG | ssc04914 Progesterone-mediated oocyte maturation | CPEB1, IGF1R |
| KEGG | ssc00640 Propanoate metabolism | ACACA, PCCB |
| KEGG | ssc05215 Prostate cancer | FOXO1, IGF1R, MDM2, MTOR |
| KEGG | ssc04974 Protein digestion and absorption | COL11A1, COL14A1 |
| KEGG | ssc04141 Protein processing in endoplasmic reticulum | DNAJC3, MAN1A2, NPLOC4, PLAA, SSR3, STT3B |
| KEGG | ssc05205 Proteoglycans in cancer | FZD4, IGF1R, MDM2, MTOR, RAC3, TIAM1 |
| KEGG | ssc00230 Purine metabolism | AK7, PDE8A, POLR3C, |
| KEGG | ssc00620 Pyruvate metabolism | ACACA, ME1 |
| KEGG | ssc04015 Rap1 signaling pathway | IGF1R, RAC3, RAPGEF1, TIAM1 |
| KEGG | ssc04014 Ras signaling pathway | IGF1R, PAK5, RAC3, RALBP1, TIAM1 |
| KEGG | ssc04810 Regulation of actin cytoskeleton | PAK5, RAC3, TIAM1, |
| KEGG | ssc04140 Regulation of autophagy | ATG7, IFNG |
| KEGG | ssc05211 Renal cell carcinoma | PAK5, RAC3, RAPGEF1, |
| KEGG | ssc05323 Rheumatoid arthritis | IFNG, TNFRSF11A |
| KEGG | ssc03018 RNA degradation | DCP1A, DIS3 |
| KEGG | ssc04970 Salivary secretion | BST1, CD38 |
| KEGG | ssc05132 Salmonella infection | IFNG, PKN2, RAC3, |
| KEGG | ssc04726 Serotonergic synapse | HTR3B, HTR4 |
| KEGG | ssc04550 Signaling pathways regulating pluripotency of stem cells | FZD4, IGF1R, MYF5 |
| KEGG | ssc04721 Synaptic vesicle cycle | CPLX4, UNC13C |
| KEGG | ssc04660 T cell receptor signaling pathway | IFNG, NCK1, PAK5, |
| KEGG | ssc04350 TGF-beta signaling pathway | BAMBI, IFNG |
| KEGG | ssc04919 Thyroid hormone signaling pathway | FOXO1, MDM2, MTOR, SLCO1C1, THRB |
| KEGG | ssc05202 Transcriptional misregulation in cancer | ETV6, FOXO1, IGF1R, MDM2 |
| KEGG | ssc05152 Tuberculosis | CLEC7A, IFNG, PLA2R1, |
| KEGG | ssc04120 Ubiquitin mediated proteolysis | MDM2, UBE3A |
| KEGG | ssc05203 Viral carcinogenesis | MDM2, RAC3, UBE3A |
| KEGG | ssc04310 Wnt signaling pathway | BAMBI, FZD4, RAC3 |
